# Erratum: Jiang et al., “A Preprocessing Toolbox for 2-Photon Subcellular Calcium Imaging”

**DOI:** 10.1523/ENEURO.0290-25.2025

**Published:** 2025-08-19

In the article “A Preprocessing Toolbox for 2-Photon Subcellular Calcium Imaging,” by Anqi Jiang, Chong Zhao, and Mark E. J. Sheffield which was published online on May 13, 2025, Anqi Jiang and Chong Zhao should be listed as co-first authors. The online version has been corrected to include the following statement as a footnote: “A.J. and C.Z. equally contributed as the co-first authors.”

